# Prognostic significance of pretreatment serum free fatty acid in patients with diffuse large B-cell lymphoma in the rituximab era: a retrospective analysis

**DOI:** 10.1186/s12885-021-08963-6

**Published:** 2021-11-21

**Authors:** Liping Fan, Qiuyan Lin, Xiaoling Huang, Danhui Fu, Haobo Huang

**Affiliations:** 1 Department of Blood Transfusion, Fujian Medical University Union Hospital, Gulou District, Fuzhou City, 350001 Fujian Province China; 2 Fujian Institute of Hematology, Fujian Provincial Key Laboratory on Hematology, Department of Hematology, Fujian Medical University Union Hospital, Gulou District, Fuzhou City, 350001 Fujian Province China

**Keywords:** DLBCL, Free fatty acid, Prognostic factor

## Abstract

**Background:**

Fatty acid metabolism is reportedly associated with various cancers. However, the role of pretreatment serum free fatty acid (FFA) levels in diffuse large B-cell lymphoma (DLBCL) prognosis is still unclear, and our study aimed to better elucidate its influence on clinical outcomes.

**Methods:**

The medical records of 221 newly diagnosed DLBCL patients admitted to Fujian Medical University Union Hospital from January 2011 to December 2016 were analysed retrospectively. Receiver operating characteristic curve analysis was used to determine a cut-off value for pretreatment serum FFA levels for prognostic prediction in DLBCL patients. The relationship between pretreatment serum FFA levels and clinical and laboratory parameters was analysed. Univariate and multivariate analyses were used to assess prognostic factors for overall survival (OS) and progression-free survival (PFS).

**Results:**

Newly diagnosed DLBCL patients with high pretreatment serum FFA levels (≥0.495 mmol/l) had more B symptoms, higher serum lactate dehydrogenase levels (> upper limit of normal), >1 extranodal site, and higher International Prognostic Index score (3–5) compared to those with low pretreatment serum FFA levels (<0.495 mmol/l). Higher serum FFA levels were independent prognostic factors for poor OS, but not PFS.

**Conclusions:**

High pretreatment serum FFA levels are associated with lower survival in untreated DLBCL patients.

## Background

Diffuse large B-cell lymphoma (DLBCL) is the most common type of B-cell non-Hodgkin’s lymphoma (NHL), accounting for 30-40% of all NHLs worldwide. Currently, the first-line regimen is the anti-CD20 monoclonal antibody rituximab plus cyclophosphamide, doxorubicin, vincristine, and prednisone. However, the heterogeneity in immunology, cytogenetics, and molecular biology of DLBCL patients lead to diverse clinical outcomes. Moreover, 40-50% of the patients suffer from relapsed or refractory DLBCL [1–3]. Therefore, treatment should be based on risk stratification.

The earliest prognostic index for NHL was the International Prognostic Index (IPI) score, which was established before the wide application of rituximab in clinical practice and had been based on clinical factors, including age, Ann Arbor stage, extranodal disease, Eastern Cooperative Oncology Group (ECOG) score, and serum lactate dehydrogenase (LDH) level. The addition of rituximab to the standard chemotherapy regimen has dramatically improved the outcomes of DLBCL patients; however, several studies showed that the IPI could not effectively predict the prognosis of DLBCL patients. Since then, the revised IPI and the National Comprehensive Cancer Network IPI (NCCN-IPI) were generated [4, 5]. Nevertheless, none of these indexes could precisely predict DLBCL patient prognosis [6, 7]. Therefore, it is necessary to identify other factors in patients with DLBCL as predictors of prognosis.

Abnormal lipid metabolism is considered to be an important feature of tumor development and progression [8, 9]. Some researchers demonstrated that abnormal lipid metabolism occurred in DLBCL patients as well as in cell lines [10, 11]. Free fatty acids (FFAs) are an important energy source for cells and bind to albumin in the blood. Some studies showed that abnormal serum FFA levels were associated with poor prognosis in several types of malignancy, such as prostate cancer, lung cancer, gastric cancer, thyroid cancer, colorectal cancer, and ovarian cancer [12, 13]. However, the association between serum FFA levels and DLBCL is still unclear. Therefore, we retrospectively analysed the data of DLBCL patients in our hospital, intending to evaluate the prognostic value of pretreatment serum FFAs.

## Methods

### Patient selection

This study was conducted in accordance with the Declaration of Helsinki (as revised in 2013). The study design was approved by the Ethics Committee of Fujian Medical University Union Hospital. As this study was a retrospective data analysis and did not affect patients’ treatments, written informed consent was not sought.

**Fig. 1:**
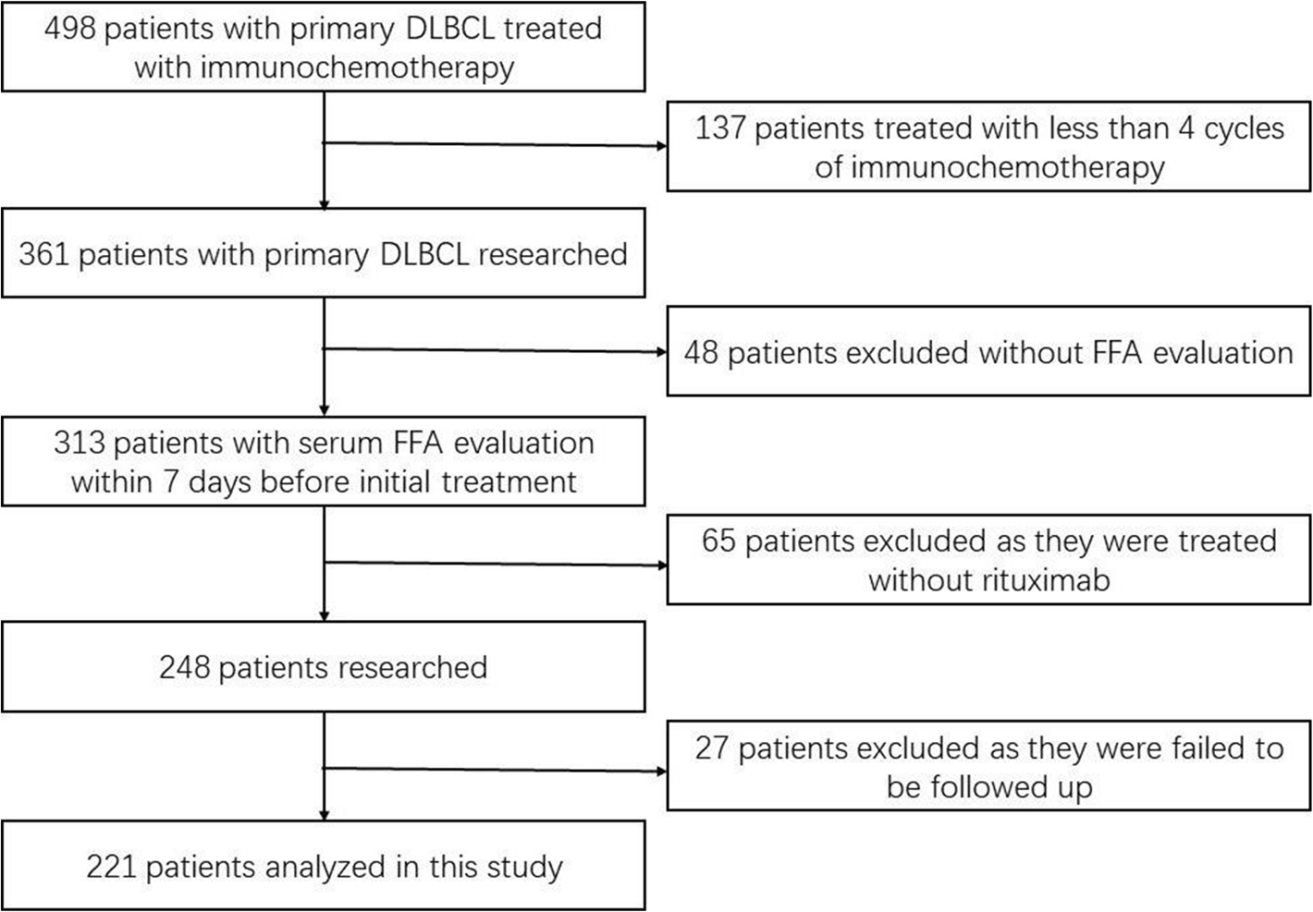
A flowchart showing retrospective screening for DLBCL patients

We studied 361 newly diagnosed DLBCL patients who were admitted to Fujian Medical University Union Hospital from January 1, 2011, to December 31, 2016. Patients who were ≥14 years old and received ≥4 cycles of immunochemotherapy were included in the study. All patients were diagnosed according to the World Health Organization classification. Patients diagnosed with primary mediastinal lymphoma, primary central nervous system lymphoma, human immunodeficiency virus infection, or diabetes mellitus were excluded. Clinical and laboratory parameters, including age, sex, B symptoms, serum LDH levels, ECOG score, Ann Arbor stage, extranodal disease sites, IPI score, cell of origin, serum FFAs levels, and immunochemotherapy regimens, were collected from medical records. Based on the screening flowchart depicted in Fig.1, a total of 221 patients were included in the analysis.

### Sample detection

Peripheral blood samples were collected from all patients within a week before the primary therapy. Peripheral blood samples collected from 221 healthy individuals without metabolic and neoplastic diseases, presenting in the same time period, sex- and age-matched to the DLBCL cases were designated as the normal control group. All samples were collected from the patients and healthy individuals after a night of fasting.

The serum FFAs that we measured were non-esterified fatty acids. FFAs were detected by the enzymatic endpoint method using an automatic biochemical analyser according to the manufacturer’s instructions. Detection reagents were purchased from FUJIFILM Wako Pure Chemical Corporation (Japan).

### Treatment and follow-up

The 221 patients included in this study had received ≥4 cycles of immunochemotherapy consisting of cyclophosphamide, doxorubicin or epirubicin, vincristine, prednisone, and rituximab, with or without a history of tissue biopsy or surgical excision.

Treatment response evaluation was performed according to the International Working Group Response Criteria for Malignant Lymphoma through laboratory tests and imaging examinations with or without bone marrow biopsy [14]. When disease progression or relapse occurred, patients were treated with later-line regimens recommended by the NCCN guidelines [15–17].

Overall survival (OS) was defined as the time from the date of diagnosis to the date of death due to any cause or the last follow-up. Progression-free survival (PFS) was defined as the time from the date of diagnosis to the date of disease progression, relapse, or death, whichever came first. The last date of follow-up was June 10, 2019. Follow-up data were obtained from clinical records or by telephone interviews with the patients or their relatives.

### Statistical analyses

All statistical analyses were performed using SPSS version 19.0 software (SPSS, Inc. Chicago, IL, USA) for Windows. The receiver operating characteristic (ROC) curve was used to determine the optimal cut-off value for FFAs. Serum FFAs levels were compared to clinical and laboratory parameters using the Chi-square test. The Kaplan-Meier method was applied for the analysis of time-to-event data. The Cox proportional hazard model was used for the univariate analysis of potential survival predictors. The stepwise forward Cox regression model was used for multivariate analysis of statistically significant clinical variables determined from the univariate analysis. The Log-rank test was used to compare the survival time of the different groups. A two-sided *P* value <0.05 was considered statistically significant.

## Results

### Patients’ characteristics

From January 2011 to December 2016, 221 patients with newly diagnosed DLBCL who met the inclusion criteria were enrolled in this study (Table 1). This cohort consisted of 128 (57.92%) males and 93 (42.08%) females. The median age at diagnosis was 54 years (range: 14–86), with 72 (32.58%) patients being >60 years old. In this cohort, 57 (25.79%) patients had B symptoms, 102 (46.15%) had higher (>upper limit of normal) LDH levels, 50 (22.62%) had high ECOG scores (2-4), and 139 (62.90%) had advanced cancer stages (III-IV). The number of patients with IPI scores corresponding to low-risk (0–1), low-intermediate-risk (2), high-intermediate risk (3), and high-risk (4–5) were 88 (39.82%), 54 (24.44%), 50 (22.62%), and 29 (13.12%), respectively. The diagnostic tissue samples of 23 patients were insufficient to determine the cell of origin. The cell of origin for 76 (34.39%) patients was the germinal center B-cell (GCB), and 122 (55.20%) patients were non-GCB.

**Table 1:** Demographic and clinical parameters of enrolled patients(*n*=221)

Parameters	Classification	FFAs		P
		Low(*n*=106)	High(*n*=115)	
Age (years)	>60	32 (30.19%)	40 (34.78%)	0.477
	≤60	74 (69.81%)	75 (65.22%)	
Sex	Male	65 (61.32%)	63 (54.78%)	0.343
	Female	41 (38.68%)	52 (45.22%)	
B symptoms	Absent	88 (83.02%)	76 (66.09%)	0.005
	Present	18 (16.98%)	39 (33.91%)	
LDH	>ULN	31 (29.25%)	71 (61.74%)	<0.001
	Normal	75 (70.75%)	44 (38.26%)	
ECOG score	0-1	88 (83.02%)	83 (72.17%)	0.076
	2-4	18 (16.98%)	32 (27.83%)	
Ann Arbor Stage	I-II	44 (41.51%)	38 (33.04%)	0.212
	III-IV	62 (58.49%)	77 (66.96%)	
Extranodal	> 1	23 (21.70%)	41 (35.65%)	0.026
disease	≤ 1	83 (78.30%)	74 (64.35%)	
IPI	Low risk (0-1)	52 (49.06%)	36 (31.30%)	0.008
	Low-intermediate risk (2)	28 (26.42%)	26 (22.61%)	
	High-intermediate risk (3)	16 (15.09%)	34 (29.57%)	
	High risk (4-5)	10 (9.43%)	19 (16.52%)	
Cell of origin	GCB	36 (33.96%)	40 (34.78%)	0.416
	non-GCB	56 (52.83%)	66 (57.39%)	
	Undetermined	14 (13.21%)	9 (7.83%)	

### Serum FFA levels in DLBCL patients and healthy individuals

**Fig. 2:**
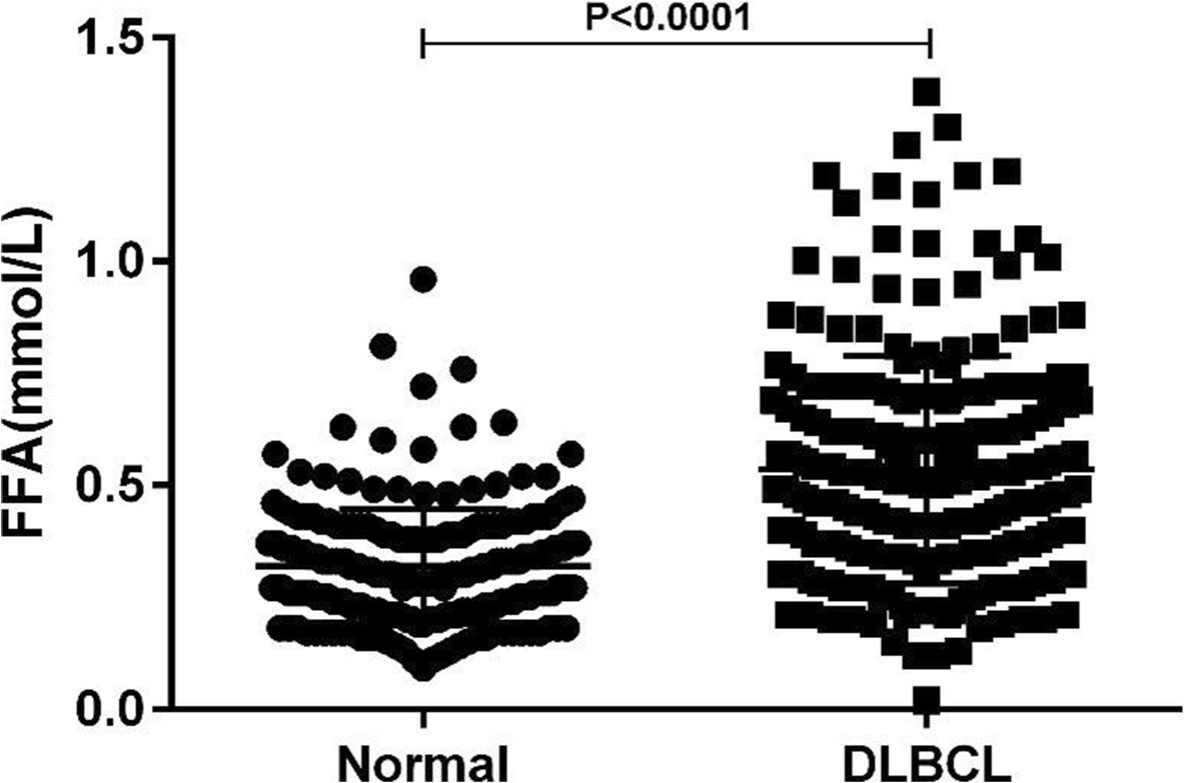
Serum FFAs level in DLBCL patients and healthy individuals

Pretreatment serum FFA levels in DLBCL patients ranged from 0.02–1.38 mmol/l with a median value of 0.51 mmol/l. Serum FFA levels in healthy individuals ranged from 0.09–0.96 mmol/l with a median value of 0.3 mmol/l. The results showed that serum FFA levels in DLBCL patients were significantly higher than those in healthy individuals (0.535 ± 0.017 vs. 0.319 ± 0.009) (Fig.2).

### Identification of optimal FFA cut-off values and patient outcomes

**Fig. 3:**
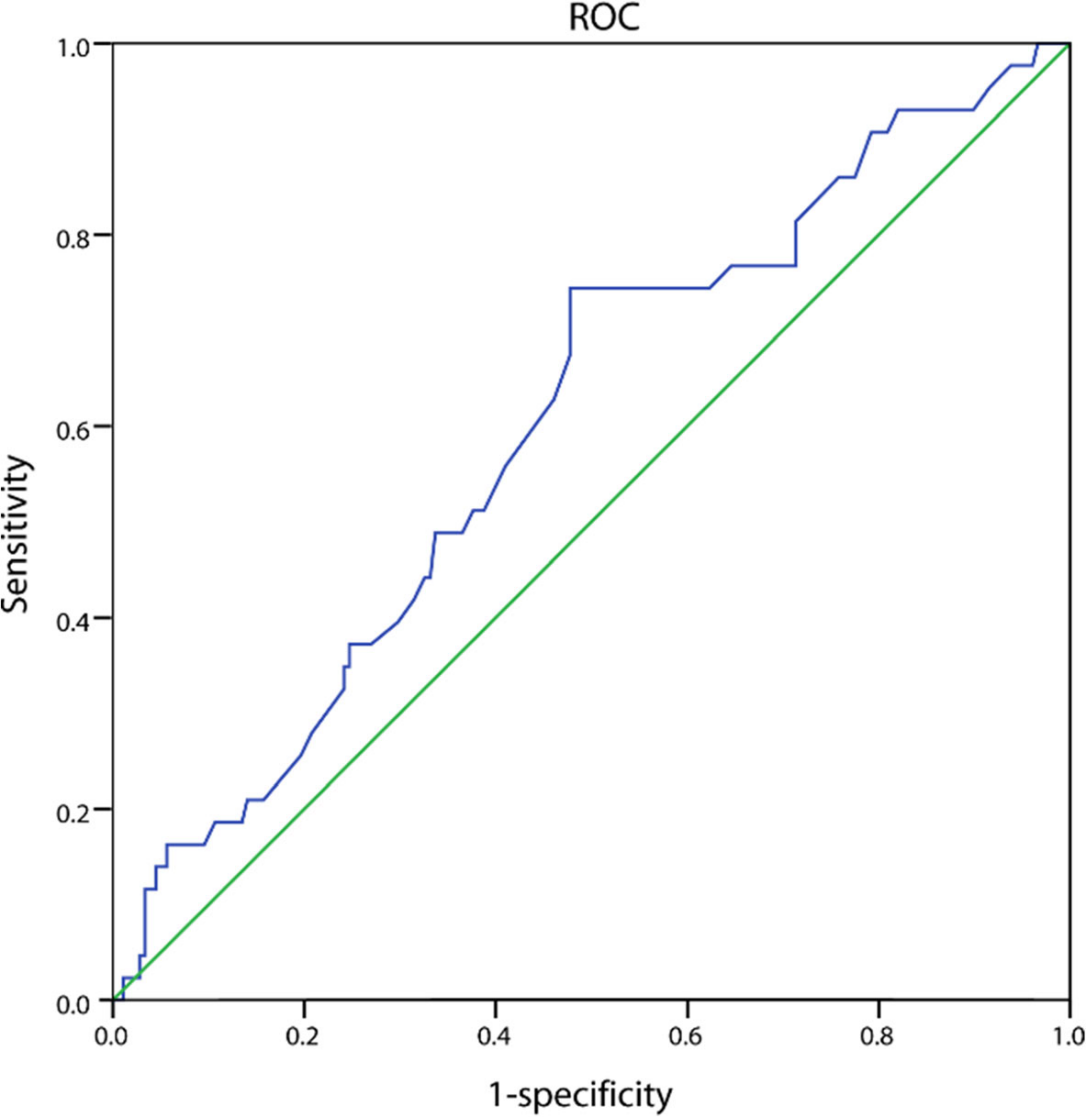
ROC curve analysis of pretreatment serum FFAs levels (AUC=0.602,95% CI 0.510-0.694, *P*=0.038)

The optimal FFA cut-off value determined by the ROC curve was 0.495 mmol/l, with an area under the curve value of 0.602 (95% CI, 0.510–0.694, *P*=0.038) (Fig.3). In this cohort, 115 (52.04%) patients were assigned to the high FFAs group and 106 (47.96%) to the low FFAs group.

The clinical and laboratory parameters, as well as the comparison between the high FFAs and low FFAs groups, are shown in Table 1. Patients in the high FFAs group had more B symptoms (33.91% vs. 16.98%, *P*=0.005), higher LDH level (>upper limit of normal) (61.74% vs. 29.25%, *P*<0.001), more cases of >1 extranodal site (35.65% vs. 21.70%, *P*=0.026) and higher IPI (3-5) (46.09% vs. 24.52%, *P*=0.007) than those in the low FFAs group. There were no statistically significant differences in age, sex, ECOG score, Ann Arbor stage, and cell of origin between these two groups.

### Univariate and multivariate analyses of potential prognostic factors for survival

The median follow-up period of this cohort was 45.03 months (range: 2.93–96.3 months), and 43 patients died. The median OS and PFS were not reached in this cohort.

**Fig. 4:**
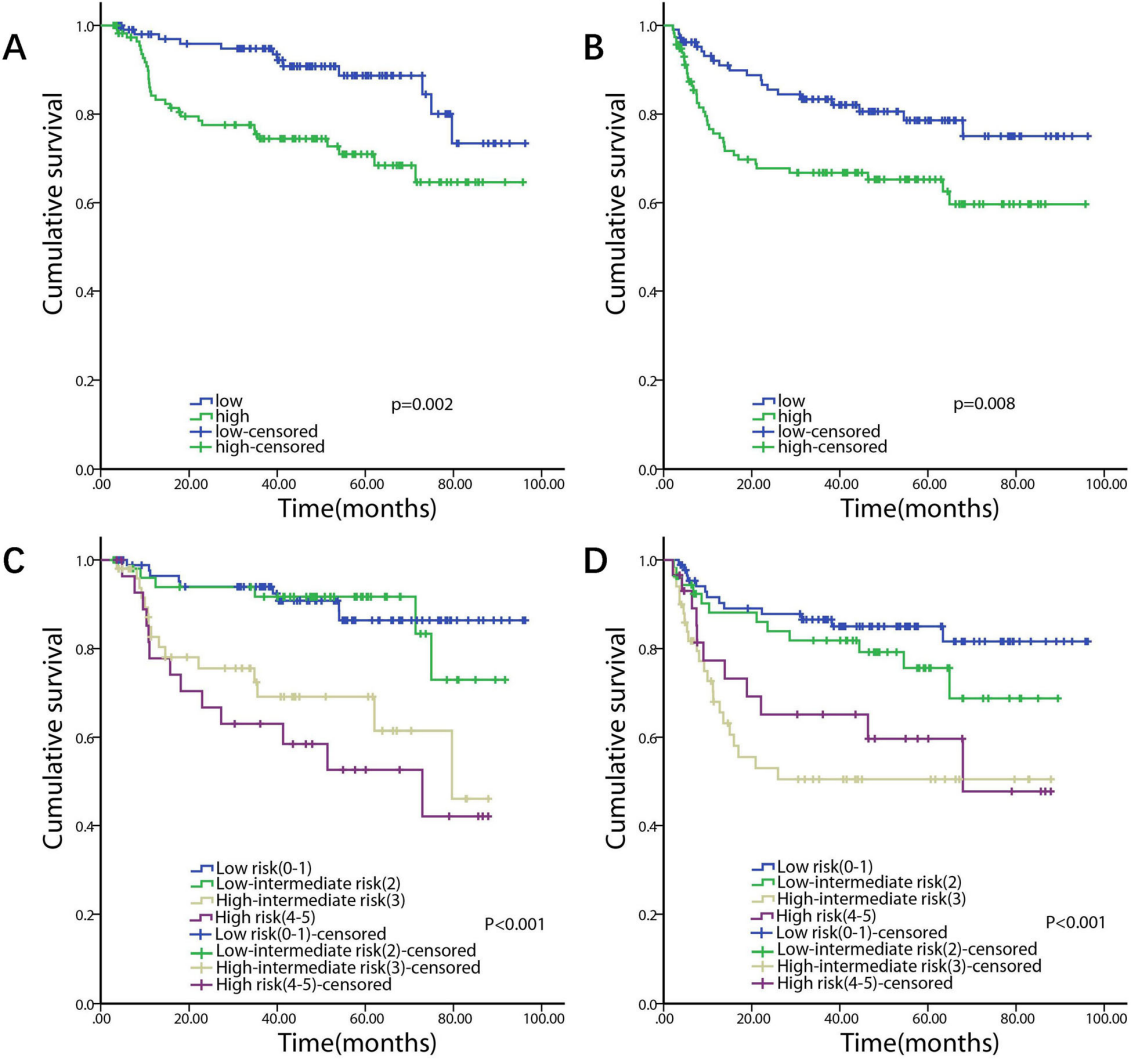
Kaplan-Meier curves of survival with respect to pretreatment serum FFAs levels (**A**: OS, **B**: PFS) and IPI scores (**C**: OS, **D**: PFS)

Univariate analysis showed that higher serum FFA levels (≥0.495 mmol/l) (*P*=0.004), as well as the presence of B symptoms (*P*=0.007), elevated LDH (*P*<0.001), ECOG score ≥2 (*P*=0.001), >1 extranodal site (*P*=0.016), and high-intermediate risk (3) or high-risk (4-5) IPI score (*P*=0.001 and *P*<0.001, respectively) were significantly associated with shorter OS. Multivariate analysis showed that higher serum FFA levels (≥0.495 mmol/l) (*P*=0.036) and high IPI score (*P*=0.001) were independent prognostic factors for shorter OS (Table 2, Fig.4).

**Table 2:** Univariate and multivariate analysis of prognostic factors for OS in patients with DLBCL

Parameters	Univariate analysis	Multivariate analysis		
	HR (95% CI)	P	HR (95% CI)	P
Age (>60 vs ≤60)	1.567 (0.850–2.891)	0.150		
Sex (male vs female)	1.008 (0.550–1.848)	0.980		
B symptoms (present vs absent)	2.302 (1.255–4.221)	0.007		
LDH (>ULN vs normal)	4.022 (2.026–7.984)	<0.001		
ECOG score (2-4 vs 0-1)	2.785 (1.518–5.108)	0.001		
Ann Arbor Stage (III-IV vs I-II)	1.919 (0.967–3.810)	0.062		
Extranodal disease (>1 vs ≤1)	2.089 (1.144–3.815)	0.017		
IPI		<0.001		<0.001
Low risk (0-1)	reference		reference	
Low-intermediate risk (2)	1.146 (0.408–3.219)	0.797	1.144 (0.407–3.215)	0.798
High-intermediate risk (3)	4.044 (1.763–9.276)	0.001	3.516 (1.519–8.139)	0.003
High risk (4-5)	5.441 (2.324–12.739)	<0.001	4.722 (1.999–11.154)	<0.001
Subtype (GCB vs non-GCB)	1.254 (0.795–1.979)	0.331		
FFAs (high vs normal)	2.698 (1.385–5.258)	0.004	2.075 (1.051–4.096)	0.035

On the other hand, univariate analysis showed that higher serum FFA levels (≥0.495 mmol/l) (*P*=0.009), as well as elevated LDH (*P*=0.003), ECOG score ≥2 (*P*=0.007), advanced Ann Arbor Stage (III-IV) (*P*=0.01), >1 extranodal site (*P*=0.006), and high-intermediate risk (3) or high-risk (4-5) IPI score (both *P*<0.001) were significantly associated with shorter PFS. Multivariate analysis showed that high IPI score (*P*=0.001) was an independent prognostic factor for shorter PFS (Table 3, Fig.4).

**Table 3:** Univariate and multivariate analysis of prognostic factors for PFS in patients with DLBCL

Parameters	Univariate analysis		Multivariate analysis	
	HR (95% CI)	P	HR (95% CI)	P
Age (>60 vs ≤60)	1.282 (0.745–2.204)	0.370		
Sex (male vs female)	0.927 (0.552–1.555)	0.773		
B symptoms (present vs absent)	1.421 (0.815–2.480)	0.216		
LDH (>ULN vs normal)	2.260 (1.329–3.843)	0.003		
ECOG score (2-4 vs 0-1)	2.111 (1.228–3.630)	0.007		
Ann Arbor Stage (III-IV vs I-II)	2.253 (1.214–4.179)	0.010		
Extranodal disease (>1 vs ≤1)	2.063 (1.226–3.472)	0.006		
IPI		<0.001		<0.001
Low risk (0-1)	reference		reference	
Low-intermediate risk (2)	1.592 (0.726–3.490)	0.245	1.592 (0.726–3.490)	0.245
High-intermediate risk (3)	4.238 (2.127–8.444)	<0.001	4.238 (2.127–8.444)	<0.001
High risk (4-5)	3.140 (1.406–7.012)	0.005	3.140 (1.406–7.012)	0.005
Subtype (GCB vs non-GCB)	1.151 (0.774–1.711)	0.487		
FFAs (high vs normal)	2.050 (1.193–3.525)	0.009		

## Discussion

Outcomes after the first-line treatment vary for DLBCL patients; therefore, precise risk stratification is important for the choice of treatment and prognosis. However, The IPI and its derivatives are imprecise in predicting the overall outcome of DLBCL patients [4–7]. Hence, identifying specific and universal clues related to cancer development and progression is crucial to help construct useful prognostic indexes.

Metabolic reprogramming is one of the hallmarks of cancer. Metabolism-related genes, processes, and metabolites have essential roles in cancer development and progression [8, 9]. Abnormal lipid metabolism has a significant association with the progression of hematological and other malignancies [10, 11, 18–24]. Furthermore, statins have demonstrated synergistic antitumor effects *in vitro* and have improved the survival of patients with several types of cancer, such as DLBCL, bladder cancer, breast cancer, lung cancer, adrenocortical cancer, and T-lymphoma [25–31]. Therefore, studies focusing on the important molecules or pathways related to lipid metabolism will be helpful in the development of new DLBCL diagnostic biomarkers or therapeutic targets.

FFAs, also named non-esterified fatty acids, largely originate from the lipolysis of triacylglycerol stored in the adipose tissue. These fatty acids are composed mainly of oleic acid, palmitic acid, and linoleic acid. In cells, fatty acids accompanied by fatty acid-binding proteins (FABPs) are transferred to some compartments to function, such as mitochondria and peroxisome for β-oxidation, endoplasmic reticulum for trafficking and signaling and membrane synthesis, and nucleus to regulate the transcription of genes such as PPAR, FABPs, VEGF and PDK1. Therefore, FFAs can act as sources of energy, substrates for membrane structures, and precursors of several intracellular signaling molecules in cells [32, 33]. Recent researches have shown that fatty acid metabolism has an important role in determining the fate of cancer cells.

Fatty acid metabolism in humans is affected by many factors, including endocrine metabolic diseases, diet, and pregnancy. Zhang et al. have reported serum FFA levels with a median value of 0.41 mmol/L (IQR 25^th^–75^th^, 0.29–0.56) in the normal population [13]. This result differed from our finding, probably because fatty acid metabolism varies according to age, and the age range of the normal population in our study (14–86 years) differed from that in the study by Zhang et al. (18–65 years). Abnormal serum FFA levels were usually found in patients with metabolic diseases, such as diabetes and hyperthyroidism, and more recently, in association with some malignancies [10, 11, 18–24]. Studies that have uncovered the relationship between FFAs and cancer also identified the cut-off value of serum FFAs according to cancer diagnosis [12, 13, 34]. However, it should be noted that the cut-off values of serum FFAs in different studies or different cancer types are varied. Furthermore, few studies have been reported about the relationship between pretreatment serum FFAs levels and DLBCL development and progression. Although Thanarajasingam et al. found that low levels of plasma Omega-3 fatty acid, a kind of FFA, were associated with inferior event-free survival in untreated DLBCL patients [35], our impression was that the prognostic efficacy of serum Omega-3 fatty acid levels in DLBCL patients was limited due to the small sample size of the study. Therefore, we sought to investigate serum FFAs levels and their association with the prognosis of untreated DLBCL patients in a larger DLBCL patient population.

The main characteristics of patients included in our study were similar to previous studies. However, the median age of our cohort was 54 years, which was lower than that in other studies. The main cause of this difference could be that ≥14-year-old adolescent patients enrolled in our study received the same therapeutic regimens as adult patients. Interestingly, we found that serum FFA levels in DLBCL patients were higher than those in healthy individuals. To explore the prognostic value of serum FFAs in patients with newly diagnosed DLBCL, we first identified the optimal cut-off value of pretreatment serum FFA levels according to the patients’ survival. Then, we analyzed the relationships between pretreatment serum FFA levels and the clinical and laboratory parameters of patients with newly diagnosed DLBCL. Our data showed that DLBCL patients with high pretreatment serum FFA levels had more B symptoms, higher serum LDH levels, more extranodal sites, and higher IPI scores than those with low pretreatment serum FFA levels. Although previous studies report that age and sex may be potential confounding factors in assessing the risk for metabolic disturbance using FFAs biomarkers [34], our results showed that there was no difference in pretreatment serum FFAs among DLBCL patients who belonged to different age or sex groups. This finding supports the possibility of using serum FFAs as a biomarker for the prognosis of DLBCL patients. On the other hand, univariate and multivariate analyses showed that IPI scores, in addition to higher pretreatment serum FFA levels, were independent poor-prognosis factors for OS in patients with newly diagnosed DLBCL. The other parameters considered to be significant factors through univariate analysis, such as ECOG score, and serum LDH levels, were components of the IPI score. Therefore, since the predictive value of these parameters had already been included in the IPI score, they could not act as independent prognostic factors for OS in patients with newly diagnosed DLBCL.

Nevertheless, we would like to acknowledge some limitations in our study. The number of patients included in our study was insufficient for division into two sets, a training set and a validation set, to confirm our conclusions. Furthermore, the cut-off value of pretreatment serum FFAs from our single-centre study may not be applicable for analysis in other centres; it is necessary to set up a universal cut-off value for DLBCL patients from different centres. Moreover, we detected more than a single kind of FFA. This may be the reason for the relatively low sensitivity and specificity of FFAs. Further investigation is required to distinguish the kinds of FFAs that are associated with the prognosis of DLBCL patients, and to determine the specific metabolic pathways involved in this pathophysiological process. Finally, although patients with diabetes mellitus were excluded from our study, patients with impaired glucose tolerance were included. This abnormal metabolism might have introduced a potential bias in our analysis. Based on the limitations described above, a multicentre, prospective, and larger study is needed to discover and validate novel lipid metabolism-related molecular markers or pathways in DLBCL development and progression.

## Conclusions

We demonstrated the clinical significance of pretreatment serum FFA levels in patients with newly diagnosed DLBCL. Our results indicated that a high pretreatment serum FFA level was associated with adverse features such as the presence of B symptoms, higher serum LDH levels, >1 extranodal sites, and a higher IPI score. Survival analysis also demonstrated that higher pretreatment serum FFAs were associated with lower survival in untreated DLBCL patients. These findings indicate that abnormal lipid metabolism may contribute to DLBCL development and progression. Focusing on lipid metabolism will be helpful in discovering further diagnostic and therapeutic targets for patients with DLBCL.

## Data Availability

All data generated or analysed during this study are included in this published article.

## Abbreviations

DLBCL: Diffuse large B-cell lymphoma
NHL: Non-Hodgkin’s lymphoma
IPI: International Prognostic Index
ECOG: Eastern Cooperative Oncology Group
LDH: Lactate dehydrogenase
NCCN: National Comprehensive Cancer Network
FFAs: Free Fatty Acids
OS: Overall survival
PFS: Progression-free survival
ROC: Receiver operating characteristic
GCB: Germinal center B-cell
ULN: Upper limit of normal
FABPs: Fatty acid-binding proteins
